# 

**Published:** 1885-07

**Authors:** 


Vol. XXI. | July, 1885. | No. 2.

### The Bistoury.

Thad S. Up de Graff, M.D.
EDITOR.

ELMIRA, N.Y.

